# Histopathological analysis of soft tissue changes in gingival biopsied specimen from patients with underlying corona virus disease associated mucormycosis (CAM)

**DOI:** 10.4317/medoral.25050

**Published:** 2022-04-03

**Authors:** Deepak Pandiar, Pratibha Ramani, Reshma Poothakulath Krishnan, Dinesh Y

**Affiliations:** 1 ORCID ID: 0000-0002-0591-2960. MDS, PhD, Associate Professor. Department of Oral Pathology and Microbiology, Saveetha Dental College and Hospitals, Chennai, Tamil Nadu, Republic of India; 2 MDS, PhD, DNB, Professor and HOD. Department of Oral Pathology and Microbiology, Saveetha Dental College and Hospitals, Chennai, Tamil Nadu, Republic of India; 3 MDS, Senior Lecturer. Department of Oral Pathology and Microbiology, Saveetha Dental College and Hospitals, Chennai, Tamil Nadu, Republic of India; 4 BDS, PG resident. Department of Oral Pathology and Microbiology, Saveetha Dental College and Hospitals, Chennai, Tamil Nadu, Republic of India

## Abstract

**Background:**

Corona Virus Disease-2019 (COVID-19) is perhaps the disastrous medical emergencies that has ever hit globally with multiple strains. Amongst various sequelae, mucormycosis may be considered as the most debilitating one. Post COVID-19 mucormycosis is formally regarded as corona virus disease associated mucormycosis (CAM). The aim of the current paper is to present twelve cases of CAM with unique clinical presentation with a detailed histopathological correlation of the gingival biopsied material.

**Material and Methods:**

Twelve cases of CAM were included in the study who presented initially with non-purulent swelling of the gingiva. The clinic-demographic data pertaining to age, gender, location, laterality and presence of co-morbidities was collected along with histopathological examination of biopsied specimen.

**Results:**

The patients ranged from 31-65 years (mean age 47.33 years). There was a male predominance. Clearly, maxillary right gingiva was mostly affected and all cases presented with non purulent, non tender swelling of the gingiva. The incisional biopsy from the gingiva consistently showed pseudoepitheliomatous hyperplasia of the surface epithelium along with vacuolar degeneration, extensive stromal edema, massive mixed inflammatory reaction, congested blood vessels, hemorrhage and abundant multinucleated giant cells. Potassium hydroxide (10% KOH) mount served no additional diagnostic advantage. After two initial biopsies any suspected case of CAM with these features was treated with appropriate antifungal therapy and conservative excision.

**Conclusions:**

Gingival swelling with aforementioned histopathological features resembling post COVID-19 histological alterations could be alarming early signs of CAM and are candidate of prompt antifungal therapy rather than repeat biopsy for confirmation.

** Key words:**CAM, COVID-19, giant cells, gingiva, mucormycosis.

## Introduction

Corona Virus Disease-2019 (COVID-19) is perhaps the disastrous medical emergencies we have ever faced and it has globally affected each and every individual directly or indirectly. India had not yet healed completely from the first wave of COVID-19 when the cases started rising again in early 2021. With the highest number of daily reported cases on May 7, 2021, the second wave has affected India considerably (1). Despite a positive decline in the cases ever since, we still contributed to around 45% of the new cases detected worldwide. Dismally, during the third week of May, 2021, approximately 34% of the deaths globally were also contributed by India. Initially detected in India, the new delta variant of COVID-19 (B.1.617.2) has become the most dominant strain circulating in the UK too (2).

Amidst this chaos, another life threatening threat hit the country, formally designated as coronavirus disease-associated mucormycosis (CAM) (3), mainly affecting the Indian states of Gujarat, Maharashtra, Rajasthan, Andhra Pradesh, Karnataka, Haryana, Madhya Pradesh, Uttarakhand, and Delhi. The higher number of cases has been reported particularly after delta and delta plus variants were detected. Mucormycosis is an uncommon yet lethal fungal disease caused by group of fungi called mucormycetes. Provided an optimal condition they really grow at a staggering rate of 3 mm/h at 36ºC which is much higher as compared to other fungi (4). In the absence of early diagnosis and management, mucormycosis leads to marked irreversible facial destruction and even death. Into the bargain, there was an emergence of one more fungal infection, Aspergillosis (5).

Though the information regarding the pathological changes in various organs is documented in the literature, the data pertaining to COVID-19 related pathological alterations in the oral and maxillofacial region is puny. Thus, in the current paper we analyzed histopathological changes of the biopsied material from the gingiva of the patients clinically diagnosed as CAM. These patients presented with a unique clinical presentation and demonstrated no fungal hyphae in the biopsied gingival soft tissue, however, the histopathological features were identical to the changes seen in other body parts (6-9). To the best of our knowledge these features have not been previously reported.

## Material and Methods

The present prospective observational study included twelve cases attending the outpatient department of our institute. A prior approval was obtained from the institutional ethical clearance board (IHEC/SDC/FACULTY/21/OPATH/198). Prior informed consent was taken from all the patients to keep intraoral pictures provided their identity will not be revealed. All patients had a recent history of COVID-19 infection and presented with clinical signs suggestive of mucormycosis including non purulent gingival swelling, facial pain and nasal obstruction. None of the cases had palatal involvement. Inclusion criteria were: a) recent history of COVID-19 as confirmed by real time polymerized chain reaction (RT-PCR), b) presence of non specific gingival swelling in the absence of palatal involvement, c) history of immunosuppressive therapy, d) radiographic evidence of bone destruction and e) histopathological confirmation of mucormycosis.

Non COVID-19 associated cases of rhino-cerebral mucormycosis with classical signs of palatal involvement were excluded. The clinic-demographic data pertaining to age, gender, location, laterality and presence of co-morbidities was collected along with histopathological examination of biopsied specimen. The results were tabulated and presented in on Microsoft excel spreadsheet 2013. Descriptive analysis was done for demographic and clinical data.

## Results

- Clinico-demographic profile

**Figure 1:**
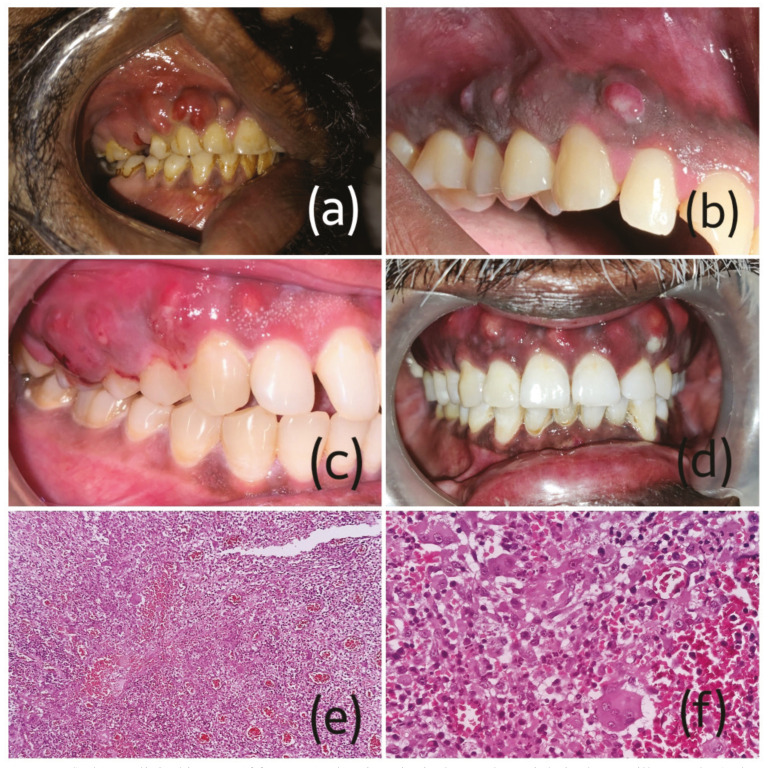
a-d) shows clinical images of four cases showing gingival growths mainly in the maxillary arch, e) photomicrograph from H&E stained sections composed of loose edematous connective tissue with rich vascularity and multinucleated giant cells (100X) and f) photomicrograph from H&E stained sections showing multinucleated cells in hemorrhagic background (400X).

In total twelve patients were included in the study. Initially, all the patients reported with clinical presentation of swelling of the attached gingiva with no pus discharge or any evidence of ulceration (Fig. 1). Interestingly, there was no sign of involvement of the palate in any of these cases. Radiographic destruction of maxillary bone was clearly evident in all cases. Table 1 shows the clinical features of all involved cases showing unique presentation. There was a definite predilection for male gender (9M:3F) with age ranging from 31-65 years (mean age-47.33 years). The maxillary gingiva of the right side was commonly affected. All cases had a history of COVID-19. Diabetes mellitus was recorded as the commonest comorbidity in eight cases. Furthermore, the patients included in the present series had no history of any previous radiotherapy or intake of bisphosphonates ruling out the possibility of medication reated osteonecrosis of jaws (MRONJ). None of the patient had history of dental extractions or any other oral surgical intervention.

**Table 1:**
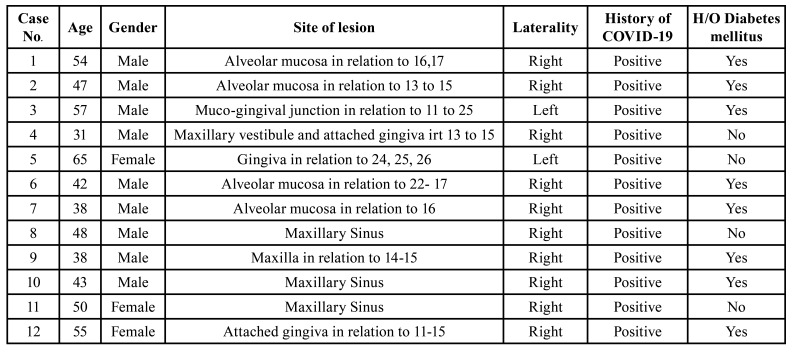
Showing clinic-demographic profile of twelve cases of CAM included in the present study.

- Histopathological analysis

An incisional biopsy from the gingival lesions of the pioneer two cases revealed hyperplastic oral epithelium with immense inflammation of underlying stroma. Fascinatingly, there were numerous multinucleated giant cells, macrophages and rich vascularity with abundant hemorrhagic areas (Fig. 1). Despite clinical and radiographical evidence of destructive bony lesions, serial sections and histochemistry (PAS staining) failed to demonstrate any fungal hyphae in the soft tissue specimen leaving no other option but to report as non specific granulomatous lesion. Final diagnosis of CAM could be made only from the second biopsy taken from the bone causing a delay in treatment.

**Figure 2:**
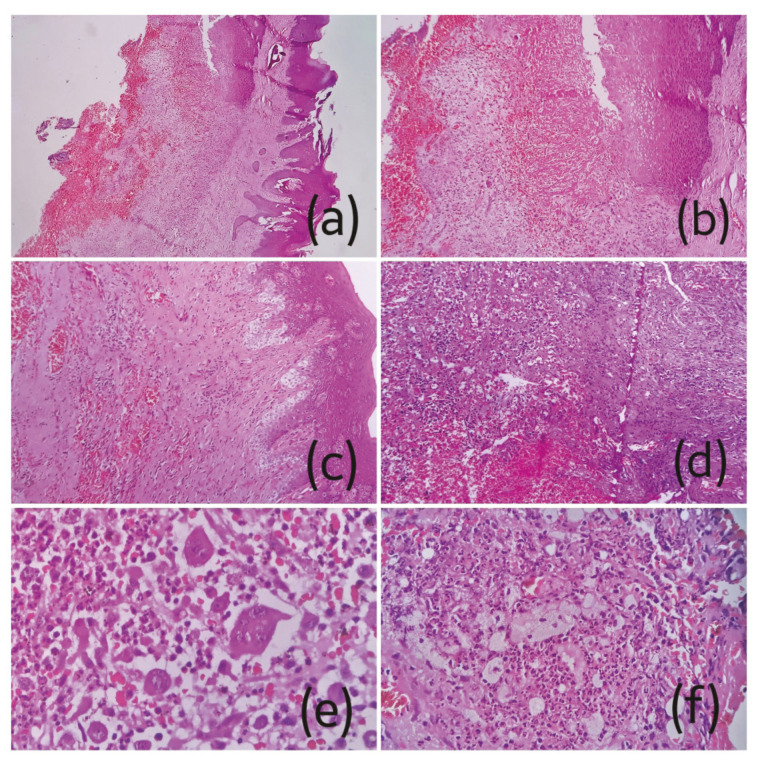
Photomicrographs of H&E stained sections showing a-c) pseudoepitheliomatous hyperplasia of epithelium along with vacuolar degeneration, extensive hemorrhage and congested vessels (a 40X, b-c 100X); d) edematous stroma with dense mixed inflammatory reaction (100X); e) numerous multinucleated giant cells in an inflammatory background (400X) and f) sheets of foamy macrophages.

**Figure 3:**
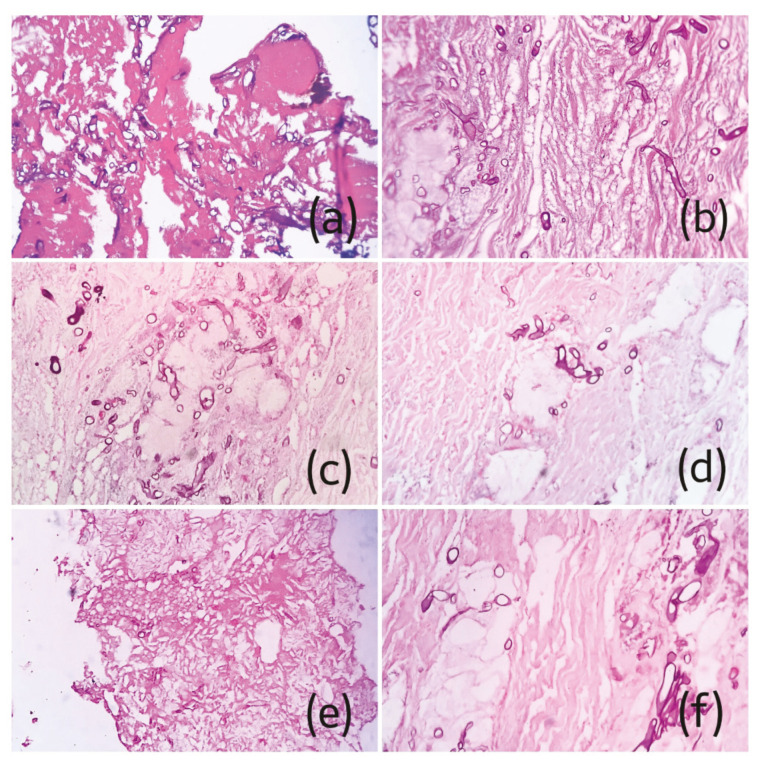
Photomicrographs of H&E stained sections showing characteristic refractile branching aseptate mucor hyphae in a necrotic background with necrotic bone. a-f ) Cases 1-6.

**Figure 4:**
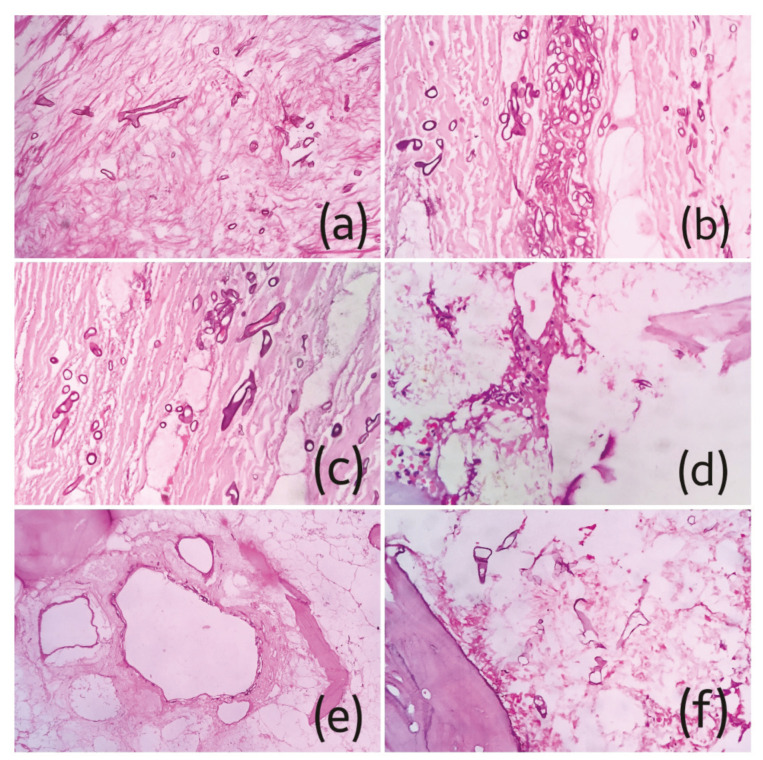
a-d,f) Photomicrographs of H&E stained sections of Case number 7-12; e) extensive vascular invasion was noted in case 11.

The features consistently noted on gingival biopsy from all cases were pseudoepitheliomatous hyperplasia of the surface epithelium along with vacuolar degeneration, extensive stromal edema, massive mixed inflammatory reaction chiefly consisting of neutrophils, histiocytes and lymphocytes, congested blood vessels, extensive hemorrhage and abundant multinucleated giant cells with the number of nuclei ranging from 2-12 (Fig. 2). 10% KOH mount was non-contributory except one case. In all twelve cases, the bone pieces showed presence of broad, aseptate, branching fungal hyphae in extensive necrotic background consistent with the diagnosis of CAM (Fig. 3, Fig. 4). Table 2 shows summary of histopathological changes in gingiva of CAM.

**Table 2:**
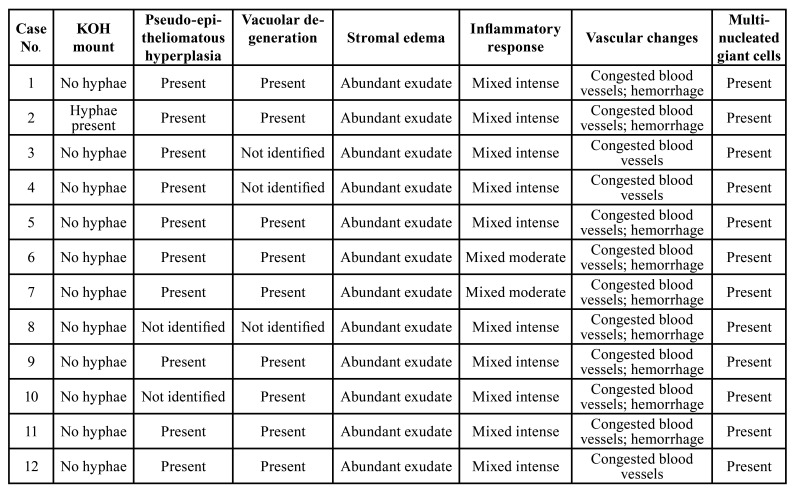
Shows summary of histopathological changes in gingiva of CAM.

In all the subsequent post-COVID-19 cases with non purulent gingival swelling, radiographic evidence of bone destruction, and presence of aforementioned histopathological features, it was thus decided to advice the operating surgeon to treat these patients with usual regimen based on availability and affordability.

- Treatment and aftermath

The cases were given Amphotericin B (1mg/kg body weight)/ lipophilic Amphotericin B (3mg/kg body weight)/ liposomal Amphotericin B (5mg/kg body weight) for 2 weeks intravenously with posaconazol orally for another 4 weeks. In cases of extensive destruction the affected bone was salvaged post antifungal therapy.Post antifungal therapy resected specimen revealed mainly degenerating fungal hyphae, extensive necrosis, dead bone and only few viable fungal hyphae. The agreement was analogous to post neoadjuvant chemotherapy related salvage of bone malignancies. This protocol led to early treatment, least disFigurement and better outcome. None of the patient developed systemic symptoms. All cases are being routinely monitored with no signs of recurrence.

## Discussion

Corona virus disease associated mucormycosis (CAM) is one amongst the other lethal sequelae of COVID-19 markedly affecting the quality of life. While the COVID-19 cases have started to decline positively, CAM patients soared in the aftermath. As aforementioned mucorales species are really fast growers, even a delay of one hour could lead to growth to as high as 3 mm (4). Thus, it is prudent to believe that any suspected case of post mucormycosis with/without comorbidities, definitive radiographic evidence of bone and gingival swellingsmay be considered as a case of CAM and thus be treated as early as possible to reduce spread to vital organs and subsequent fatalities. Here, we described post-COVID mucormycosis patients of maxillary region who presented with a unique gingival appearance. Altered taste, transient total loss of taste, aphthous stomatitis, angular cheilitis and COVID tongue are some of the common oral manifestations of corona virus disease-19 (10,11). Similarly, specific cutaneous signs have also been enumerated in literature (12,13), however, gingival signs of CAM have not been documented so far.

In the oral cavity, gingiva is unique in its structure. As formation of plaque is a continuous process, gingiva is usually chronically inflamed, which histologically shows rich capillary network and proliferating endothelium. These multiplying capillaries have a fragmented basement membrane providing easy port for micro-organisms or even tumor cells (14). Thus, gingiva could first react to any of such agent before other oral structures. Hitherto, it has established that owing to the ubiquitous presence of angiotensin converting enzyme- 2 (ACE-2) receptors, COVID-19 shows multi-system tropism including respiratory system, gastrointestinal tract (GIT), cardiovascular system (CVS), liver, nervous system, ocular system pancreas and oral structures (4). Recent studies and systematic review of literature shows histopathological changes seen in various organs but most are pertaining to the respiratory system (15,16). The pathologic findings have been attributed to direct effects of virus with characteristic inflammatory response, cytokine release, raised body temperature, inflammation, and generalized endothelial disturbance. Infection with COVID leads to atrophy and inflammation of pulmonary alveoli, vacuolar degeneration along with proliferation, desquamation and squamous metaplasia of alveolar epithelial cells (16). Other features seen are presence of monocytes and macrophages, multinucleated giant cells, extensive fibrinous exudate, and intra-cytoplasmic viral inclusion bodies (16). Similar to these changes we found pseudoepitheliomatous hyperplasia of gingival epithelium, extensive infiltration of mixed inflammatory reactions, massive edematous exudates, tissue histiocytosis and multinucleated giant cells resulting in gingival hyperplasia and an ideal environment for propagation of mucorales. Table 3 shows summary of post COVID-19 soft tissue changes in various organ systems. A comparative analysis showed that histopathological features in oral cavity most closely resembled post COVID changes seen in the lungs. Thus gingival reaction could be early sign of rhino-cerebral CAM. An additional factor that need consideration is exclusion of MRONJ as the patients with COVID-19 are at high risk to develop osteonecrosis linked to a corticosteroid-related and tocilizumab mediated risk of avascular necrosis. It is thus warranted that MRONJ must be excluded and all the patients are to be evaluated for previous oral surgical interventions (17). In all cases presented here, a diagnosis of CAM was made after histopathological confirmation.

As the leniency was provided in lockdown, the crowd in Indian markets and streets started swelling which led to anticipated early arise of third wave of COVID-19 with emergence of new strains viz Delta plus and lambda in India and other countries. Thus it is now a challenge not to just contain COVID-19 but also its sequelae including mucormycosis and Aspergillosis. It thus becomes the mandatory to screen and follow all patients healed from COVID-19 particularly with associated co-morbidities. All the patients must be adequately educated and encouraged to regularly screen their oral cavities and in case any gingival swellings are noted, the patient must self refer themselves for early diagnosis and rapid treatment of life threatening outcomes. Therefore, examination of gingiva should be considered as integral part of post COVID-19 follow-up.

**Table 3:**
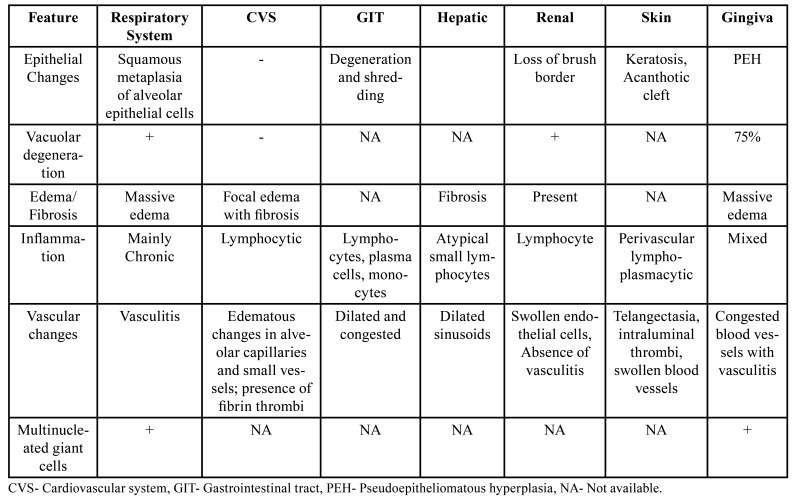
Showing post COVID-19 changes seen in other systems with comparison with oral features.

## Conclusions

As a part of post COVID-19 follow-up in any part of the world, non-purulent, non ulcerated gingival swelling in patients recovered recently from COVID-19, particularly with comorbidities and long term hospitalization/ steroidal therapy, must be considered as a candidate of CAM particularly in the presence of nonspecific giant cell granulomatous inflammation of soft tissue closely resembling the histopathological alterations seen in lungs. Such patients may be treated with early antifungal therapy and reduce the risk of progression to rhino-cerebral mucormycosis and eventually to death.

## References

[1] Raut A, Huy NT (2021). Rising incidence of mucormycosis in patients with COVID-19: another challenge for India amidst the second wave?. Lancet Respir Med.

[2] Torjesen I (2021). Covid-19: Delta variant is now UK's most dominant strain and spreading through schools. BMJ.

[3] Garg D, Muthu V, Sehgal IS, Ramachandran R, Kaur H, Bhalla A (2021). Coronavirus Disease (Covid-19) Associated Mucormycosis (CAM): Case Report and Systematic Review of Literature. Mycopathologia.

[4] Pandiar D, Kumar NS, Anand R, Kamboj M, Narwal A, Shameena PM (2021). Does COVID 19 generate a milieu for propagation of mucormycosis?. Med Hypotheses.

[5] Lai CC, Yu WL (2021). COVID-19 associated with pulmonary aspergillosis: A literature review. J Microbiol Immunol Infect.

[6] Tian S, Xiong Y, Liu H, Niu L, Guo J, Liao M (2020). Pathological study of the 2019 novel coronavirus disease (COVID-19) through postmortem core biopsies. Mod Pathol.

[7] Su H, Yang M, Wan C, Yi LX, Tang F, Zhu HY (2020). Renal histopathological analysis of 26 postmortem findings of patients with COVID-19 in China. Kidney Int.

[8] Yang M, Chen S, Huang B, Zhong JM, Su H, Chen YJ (2020). Pathological Findings in the Testes of COVID-19 Patients: Clinical Implications. Eur Urol Focus.

[9] Gianotti R, Zerbi P, Dodiuk-Gad RP (2020). Clinical and histopathological study of skin dermatoses in patients affected by COVID-19 infection in the Northern part of Italy. J Dermatol Sci.

[10] Díaz Rodríguez M, Jimenez Romera A, Villarroel M (2022). Oral manifestations associated with COVID-19. Oral Dis.

[11] Iranmanesh B, Khalili M, Amiri R, Zartab H, Aflatoonian M (2021). Oral manifestations of COVID-19 disease: A review article. Dermatol Ther.

[12] Zhao Q, Fang X, Pang Z, Zhang B, Liu H, Zhang F (2020). COVID-19 and cutaneous manifestations: a systematic review. J Eur Acad Dermatol Venereol.

[13] Daneshgaran G, Dubin DP, Gould DJ (2020). Cutaneous Manifestations of COVID-19: An Evidence-Based Review. Am J Clin Dermatol.

[14] Pandiar D, Pattamparambath M, Kalathingal PV, Maliyekkal SP, Vijay AN (2015). Deceptive Lesions of Periodontium: A Case Series. J Clin Diagn Res.

[15] Caramaschi S, Kapp ME, Miller SE, Eisenberg R, Johnson J, Epperly G (2021). Histopathological findings and clinicopathologic correlation in COVID-19: a systematic review. Mod Pathol.

[16] Deshmukh V, Motwani R, Kumar A, Kumari C, Raza K (2021). Histopathological observations in COVID-19: a systematic review. J Clin Pathol.

[17] Amorim Dos Santos J, Normando AGC, Carvalho da Silva RL, Acevedo AC, De Luca Canto G, Sugaya N (2021). Oral Manifestations in Patients with COVID-19: A 6-Month Update. J Dent Res.

